# Discriminating early-stage diabetic retinopathy with subjective and objective perimetry

**DOI:** 10.3389/fendo.2023.1333826

**Published:** 2024-01-08

**Authors:** Faran Sabeti, Joshua P. van Kleef, Rakesh M. Iyer, Corinne F. Carle, Christopher J. Nolan, Rong Hui Chia, Ted Maddess

**Affiliations:** ^1^ Eccles Institute for Neuroscience, The John Curtin School of Medical Research, The Australian National University, Canberra, ACT, Australia; ^2^ Discipline of Optometry, Faculty of Health, University of Canberra, Canberra, ACT, Australia; ^3^ Department of Endocrinology, The Canberra Hospital, Garran, ACT, Australia; ^4^ School of Medicine and Psychology, The Australian National University, Canberra, ACT, Australia; ^5^ School of Medicine, University of Western Australia, Crawley, WA, Australia

**Keywords:** multifocal, type 2 diabetes, diabetic retinopathy, objective perimetry, subjective perimetry, multifocal methods

## Abstract

**Introduction:**

To prevent progression of early-stage diabetic retinopathy, we need functional tests that can distinguish multiple levels of neural damage before classical vasculopathy. To that end, we compared multifocal pupillographic objective perimetry (mfPOP), and two types of subjective automated perimetry (SAP), in persons with type 2 diabetes (PwT2D) with either no retinopathy (noDR) or mild to-moderate non-proliferative retinopathy (mmDR).

**Methods:**

Both eyes were assessed by two mfPOP test methods that present stimuli within either the central ±15° (OFA15) or ±30° (OFA30), each producing per-region sensitivities and response delays. The SAP tests were 24-2 Short Wavelength Automated Perimetry and 24-2 Matrix perimetry.

**Results:**

Five of eight mfPOP global indices were significantly different between noDR and mmDR eyes, but none of the equivalent measures differed for SAP. Per-region mfPOP identified significant hypersensitivity and longer delays in the peripheral visual field, verifying earlier findings. Diagnostic power for discrimination of noDR vs. mmDR, and normal controls vs. PwT2D, was much higher for mfPOP than SAP. The mfPOP per-region delays provided the best discrimination. The presence of localized rather than global changes in delay ruled out iris neuropathy as a major factor.

**Discussion:**

mfPOP response delays may provide new surrogate endpoints for studies of interventions for early-stage diabetic eye damage.

## Introduction

The incidence of type 2 diabetes (T2D) has been rising globally (1), and diabetic retinopathy is a common microvascular complication of this condition. It has been estimated that 10% of persons with diabetes for 15 years or more will develop severe visual impairment, which ultimately affects 90% of persons with diabetes (2, 3). In light of the potential vision loss in the working population, the ability to identify eyes at risk of progression in a clinical setting gains significance (4). It has now been well established by our laboratory and others that changes in visual function occur before the onset of vasculopathy (5–15), suggesting that damage to the neural retina may occur early in the progression to retinopathy, possibly identifying at-risk eyes. This has also been confirmed histologically, with degeneration of retinal glia and neurons occurring before microvascular changes (16, 17). Possible therapeutics, like fenofibrate (18, 19) or candesartan (20), might be provided if we can quantify this early damage accurately enough to manage treatment.

It has been reported that short-wavelength automated perimetry (SWAP) can identify eyes with diabetic macular oedema (DMO) and retinal vasculopathy (21, 22). Matrix perimetry has also been shown to identify functional impairment before the onset of retinopathy (23). The subjective nature of such perimetry methods gives rise to high rates of fixation losses, false positives, and false negatives (24, 25). Such problems reduce the sensitivity and specificity of these methods, lowering their diagnostic utility in the clinic. Standard perimetry also suffers poor reproducibility, related to their tiny stimuli which only test around 0.5% of the assessed visual-field area (26). Objective methods that avoid these problems have been employed, including multifocal electroretinograms (mfERGs) and visual-evoked potentials (mfVEPs) (27, 28). However, these methods require long setup times (29) and have exhibited high inter-subject variability (30). Also, the diagnostic power of these methods in diabetic persons who show no retinopathy is poor (31).

Multifocal pupillographic objective perimetry (mfPOP) measures relative change in pupil responses to many concurrently presented visual-field stimuli providing rapid, objective, and non-invasive visual field testing. The method tracks the severity of retinal dysfunction consistent with the degree of retinal vascular abnormalities in persons with diabetes (9, 11, 32–34). Previously, we have reported that per-region response delays were more informative than per-region sensitivity in persons with T2D (PwT2D), easily discriminating eyes with and without retinopathy (9, 11, 32). mfERG studies have also indicated that abnormalities in regional response delays are diagnostic (14). Delay mfPOP data were also more informative than sensitivity data in persons with type 1 diabetes who had early-stage DR (33). That study found a strong association between metabolic and tissue injury factors such as body mass index (BMI), glycosylated hemoglobin (HbA1c), and creatinine. MfPOP delays are also a good marker of progression or improvement of DMO, and better than Matrix perimetry (32, 34). A recent review of 44 functional and structural measures from 23 studies indicates that mfPOP measures are significantly better at discriminating normal controls from persons with diabetes without evidence of classical retinopathy (31).

In this study, we investigated the effects of early-stage diabetic retinopathy on visual function across the central and peripheral retina with two mfPOP stimulus methods. Secondary objectives were to determine if changes in mfPOP responses could identify severity of non-proliferative diabetic retinopathy (NPDR) among PwT2D and compare the diagnostic capacity of mfPOP against SWAP and Matrix perimetric testing. The strength of this study is the evaluation of retinal dysfunction in early stages of DR before the onset of retinal vasculopathy with a head-to-head comparison of subjective and objective measures of retinal sensitivity measured on the same patients on the same day.

## Methods

### Subjects

A total of 35 subjects (mean age ± SD, 57.5 ± 11.0 years, 14 female) with T2D were recruited from The Canberra Hospital Endocrinology Department. Exclusion criteria included best corrected visual acuity (BCVA) lower than 6/9; intraocular pressure >21 mmHg; distance refraction outside ±5D, cylinder refraction >2D; pregnancy; medications that may affect retinal sensitivity or iris function; and evidence of non-diabetes-related systemic, ocular, or neurological disease that may influence retinal responses. Ethics approval was given by the ACT Health Human Research Committee (eth.7.07.667), and the study complied with the Declaration of Helsinki. All subjects provided informed consent in writing prior to experimentation.

Relevant medical information including duration of diabetes, BMI, systolic and diastolic blood pressure, recent HbA1c, lipid profile, and estimated glomerular filtration rate (eGFR) was recorded. Additional diabetes complications testing included measurement of skin autofluorescence, as a marker of tissue advanced glycation end-product (AGE) accumulation using an AGE reader (DiagnOptics, Groningen, The Netherlands) and biothesiometer testing (Bio-Medical Instrument Company, Newbury, Ohio, USA) to assess peripheral neuropathy. Blood glucose levels (BGL) were measured by a finger-prick point-of-care test after the ophthalmic testing was finished.

All patients underwent a single eye examination including a detailed history, a series of eye tests including BCVA determined using an ETDRS logMAR chart, 24-2 SWAP SITA Fast strategy (Humphrey Field Analyzer; Carl Zeiss Meditec, Inc., Dublin, CA), frequency doubling technology (Matrix) perimetry 24-2 ZEST strategy (Carl Zeiss Meditec, Dublin, Calif.), and optical coherence tomography (Spectralis, HRA+OCT; Heidelberg Engineering, Heidelberg, Germany). Both SWAP and MATRIX perimetry tested the central 24 degrees of the visual field with 54 test stimuli presented to each eye centered on the same locations. Most tests were conducted on the same day unless reasons like fatigue suggested otherwise. When SWAP and Matrix were done on the same day, SWAP was done first as subjects generally find it more taxing. When the tests were done on different days, the order was randomized. Subjects also underwent fundus photography to determine the presence and severity of NPDR based on the Early Treatment of Diabetic Retinopathy Study (ETDRS) scoring system (35). Grading was performed independently by a single ophthalmologist who was masked to the participant’s identities. The eyes of each diabetic patient were subsequently separated into two groups: no NPDR (ETDRS 10; n = 42 eyes) and mild/moderate NPDR (ETDRS 34 or 45; n = 28 eyes).

### mfPOP stimuli and data acquisition

All subjects underwent mfPOP testing with a protype of the FDA-cleared ObjectiveFIELD Analyzer (OFA; Konan Medical USA, Irvine, CA) which produces perimetric measures of mean defects (MD), pattern standard deviations (PSD), per-region total deviations (TDs), and pattern deviations (PDs), all relative to the OFA’s normative data. These measures are like those with the same names in standard automated perimetry. The acronyms and their definitions those used on for SWAP and Matrix perimetry. Briefly, the TDs are the differences (deviations) from normative data at each visual field location. Negative decibel values indicate poor sensitivity. The PDs are the TDs with the 86th percentile of the TD values subtracted off to compensate for global biases in subject performance. The MD is the mean of the TDs, and the PSD is the standard deviation (SD) of the TDs. In fact, for the HFA and Matrix perimeters, both have spatial weights applied before the mean or SD are calculated. The weights give less emphasis to peripheral locations. The OFA has TDs (and their derived measures) for response delay, that is, the differences from normal delay at each test region.

**Figure 1:**
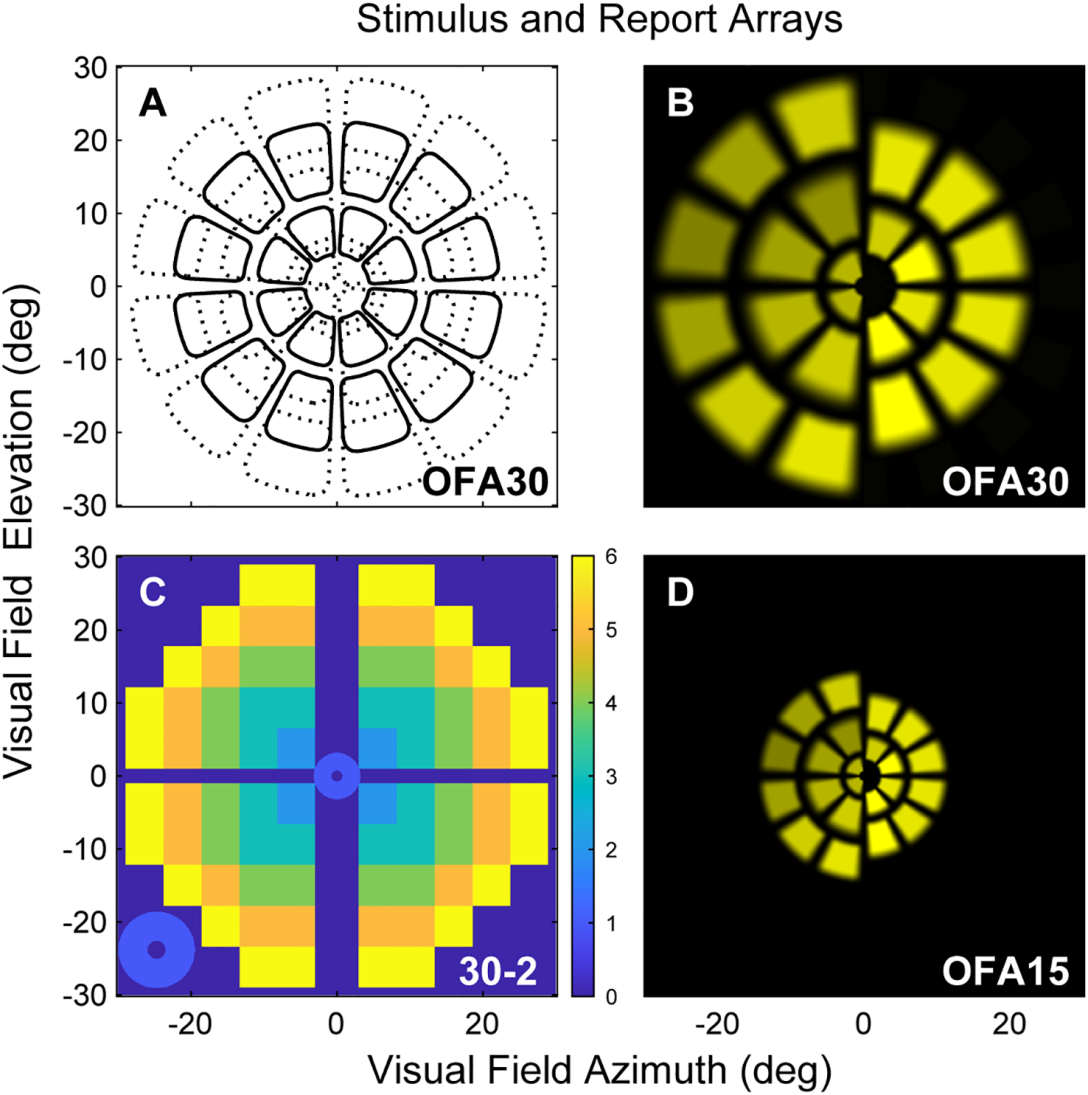
**(A)** Low luminance contours of the OFA30 stimuli showing their slightly overlapping five rings. **(B)** The left and right halves of rings 1,3,5 and 2,4 to show the luminance balancing of the test stimuli. **(C)** OFA response data, sensitivity, and delay TDs and PDs were mapped onto a 30-2 pattern with and extra four central regions to create six rings as a function of eccentric (color calibration bar at right). **(D)** Is similar to B but shows stimuli of the OFA15 method illustrating its test regions are scaled by a factor of 0.5 compared with OFA30.

To compare central to peripheral response characteristics, two OFA stimulus protocols were used to cover either the central ±30° (OFA30, **Figures 1A, B**) or ±15° (OFA15, **Figure 1D**) or of the visual field. Both protocols had yellow stimuli with a maximum luminance of 150 cd/m2 and 288 cd/m2 for OFA30 and OFA15, respectively, and were presented on 10 cd/m2 yellow backgrounds. The order of OFA protocol testing was randomized. The exact luminance of each test region was designed to elicit the same amplitude of response in a normal person, so-called luminance balancing (36). The edges of the individual stimuli were blurred to minimize the effects of mis-refraction, but trial lenses were used (37). The pseudo-randomly presented stimuli were each shown for a duration of 33 ms at an average interval of 4 s per region, yielding an aggregate presentation rate of 22/s. Each region was thus tested 90 times. Stimulus duration was 6 min in total, divided into nine segments of 40-s duration with brief resting periods between segments to minimize fatigue.

The stimuli were spatially and temporally sparse, meaning that no overlapping regions were presented simultaneously (38). A newer stimulus presentation method was employed in this study, namely, the *clustered volleys* technique, which greatly enhances the signal-to-noise ratio (39). This method has demonstrated greater power in clinical experiments compared to older mfPOP methods (40), and we have now updated our protocols to utilize these more advanced techniques.

Subjects were asked to fixate on a central red cross, which was adjusted to the patient’s habitual distance of eye deviation before each test. The two different stimulus protocols were presented dichoptically on two LCD screens at 60 frames/s. Fusion of the displays into a cyclopean view was assisted by the presentation of a dim starburst radial pattern in the background and a thin pale vertical line passing through the point of fixation. The protocols tested both eyes independently and concurrently, with both pupils recorded. Therefore, both direct and consensual responses were measured at 44 locations per visual field (**Figure 1**).

A circle was fitted in real time around the pupil to measure its diameter. Parts of the pupil extending 3 mm above the center were not measured to allow for mild ptosis. Blinks and fixation losses were detected by continuous monitoring, and any data recorded during these events was discarded. If the data loss exceeded 15%, the 40-s segment was repeated. This only occurs in around 1 in 200 tests.

### Data analysis

Data analysis was performed using MATLAB (2020b MathWorks Inc., Natick, MA). Pupil diameter was normalized to the average pupil diameter during testing for each subject. We used a non-linear regression method to estimate the pupil responses for each region from the raw pupillary waveforms (9). The per-region response amplitudes were then logarithmically transformed to decibels (dB) to stabilize the variance. Time to peak responses for each test region (per-region delays) were also recorded in milliseconds (ms). Direct and consensual responses were recorded from each eye producing 176 response estimates for each participant (2 eyes × 2 responses per region × 44 regions = 176), providing 176 per-region sensitivities and 176 delays. These were further transformed to a 30-2 pattern for reporting purposes using a method we have used before providing six rings of reported values (**Figure 1C**) (41). As discussed above, the OFA software produced summary statistics that are standard in perimetry, including MD, PSD, TDs, and PDs.

We explored effects upon OFA TDs and PDs of factors like age, sex, ETDRS severity, HbA1c levels, and visual field eccentricity. We used linear mixed effects models (the MATLAB *fitlme* function) to account for factors like the multiple regions within-eye, or eyes within subject, as required. For these models, the intercept (reference value) was the response of male subjects with ETDRS severity 10 (no retinopathy). When age was fitted, it was in decades (10 years) and was referenced to the mean age so it would not affect the intercept. Similar models examined determinants of MD and PSD data.

We compared the diagnostic performance of the perimetric tests utilizing receiver operating characteristic (ROC) analysis. For that, we took eyes of severity ETDRS 10 to be the control eyes (noDR) and examined discrimination of those eyes from eyes scored as ETDRS 34 and 45 (mild-to-moderate NPDR, mmDR). Our earlier studies of early-DR (11) have provided standardized effect sizes around 1.47. Using that and G*Power 3.1.9.7 (University of Kiel, Germany) showed that for an unequal t-test and a target p-value of 0.01, we had a power of 0.99 for a study group size of 22. For each perimeter type, the normative template was the median of the threshold values at each location across control eyes. We felt that more complex normative models that might consider factors like age and sex were not justified given our sample size. In any case, our main interest was the relative diagnostic performance of the different methods. We compared the area under the ROC curves (AUROC) calculated for each of the means of the *first-* to *twentieth-*worst deviations from normal fields for both amplitudes and delays (9). Here, we report the AUROC values for the six worst regions, since that provided close to the highest AUROC for all methods. For OFA results, we also compared discrimination of 85 matched normal controls of the OFA database (54.8 ± 12.2 years, 49 females) with eyes of the 35 PwT2D subjects (57.5 ± 11.0 years, 14 females).

## Results

### Participant data

A summary of the subject demographics is presented in **Table 1**. Between diabetes patient subgroups (noDR and mmDR), the only significant demographic difference was duration of diabetes. There was no significant difference in blood test parameters, biomarkers of diabetic tissue damage (eGFR, AGE, Biothesiometry), or basic optical coherence tomography parameters like the mean macular thickness or retinal nerve fiber layer (RNFL) thickness.

**Table 1:** Clinical and demographic information of the persons with diabetes (mean ± SD).

Measure	No NPDR OU ETDRS 10	Mild/Mod NPDR OD or OS ETDRS 34, 45
Subjects	18	17
Age (years)	55.6 ± 11.6	59.6 ± 10.2
Sex (% male)	14/18 (77)	7/17 (41)
Duration of diabetes (years)	10.5 ± 6.2	18.1 ± 9.0*
HbA_1C_ current (mmol/mol)	8.3 ± 1.7	8.7 ± 1.9
HbA_1C_ 5-year mean (mmol/mol)	8.2 ± 1.2	8.9 ± 1.7
BMI	34.3 ± 4.9	31.4 ± 5.5
Systolic BP	133.9 ± 17.1	130.9 ± 12.1
Diastolic BP	81.3 ± 11.0	76.4 ± 9.9
Total cholesterol (mmol/L)	4.12 ± 0.93	4.73 ± 0.9
Triglycerides (mmol/L)	2.25 ± 1.43	2.59 ± 1.4
HDL-cholesterol	1.09 ± 0.4	1.04 ± 0.9
LDL-cholesterol	2.09 ± 0.8	2.39 ± 1.1
Biothesiometry	16.8 ± 12.4	22.4 ± 14.5
AGE reading	2.60 ± 0.7	2.80 ± 0.6
BVCA (LogMAR)	0.00 ± 0.1	0.10 ± 0.2
OCT central 1 mm Macular thickness (µm)	280 ± 27.1	279 ± 31.2
OCT peripapillary Mean RNFL thickness (µm)	0.94 ± 10.4	1.01 ± 22.9

*Refers to statistically significant difference (p < 0.05) between DR groups.

### Mean defects and pattern standard deviations

**Table 2** examines the mean defect (MD) and pattern standard deviations (PSD) for each perimetry test. There were more values for OFA given the two tests, OFA15 and OFA30, had MD and PSD data for both sensitivities and delays. We examined differences using unpaired t-tests. The only significant differences were for both the OFA15 and OFA30 sensitivities and delays.

**Table 2:** Mean defect (MD) and pattern standard deviations (PSD) of the eyes of the PwT2D (mean ± SD).

	No NPDR ETDRS 10	Mild/Mod NPDR ETDRS 34 or 45
Eyes	42	28
Matrix MD (dB)	−0.63 ± 2.7	−1.12 ± 3.0
Matrix PSD (dB)	2.88 ± 0.9	3.00 ± 0.8
SWAP MD (dB)	−2.37 ± 3.8	−2.44 ± 3.7
SWAP PSD (dB)	3.19 ± 0.7	3.31 ± 1.0
OFA OFA15 Sensitivity MD (dB)	−6.52 ± 7.7	−10.7 ± 9.0*
OFA OFA30 Sensitivity MD (dB)	−4.46 ± 7.7	−9.19 ± 6.3**
OFA OFA15 Sensitivity PSD (dB)	6.70 ± 1.4	6.52 ± 1.6
OFA OFA30 Sensitivity PSD (dB)	6.60 ± 1.6	6.28 ± 1.7
OFA OFA15 Delay MD (ms)	39.8 ± 27.4	59.9 ± 7.4*
OFA OFA30 Delay MD (ms)	46.3 ± 28.8	70.7 ± 24.5**
OFA OFA15 Delay PSD (ms)	29.4 ± 27.5	27.5 ± 7.4
OFA OFA30 Delay PSD (ms)	27.9 ± 5.84	34.1 ± 15.8*

Statistically significant differences between DR groups: *p ≤ 0.02, **p ≤ 0.002.

We used the demographic data of **Table 1** to examine which of those variables might determine OFA mean defects (MDs) using multivariate mixed effect models (fitting eyes within subjects). We examined both the OFA for sensitivity and delay data. The results for OFA15 and OFA30 were very similar, so we only present the model data for OFA30 variables reporting some significant independent effects (**Table 3**). Only BGL and ETDRS 43 were significant for sensitivity MDs. For delay MDs, the BGL on the day, 5-year mean HbA1c, BMI, and biothesiometry were significant. Biothesiometry was not significant for the OFA15 data.

**Table 3:** Demographic variables determining OFA30 sensitivity and delay mean defect (MD) data.

*A. Sensitivity MDs (db)*				
*Name*	Estimate	SE	t-stat	p-value
*(Intercept)*	−8.05	6.073	−1.33	0.190
*ETDRS 35*	−2.71	2.155	−1.26	0.214
*ETDRS 43*	−7.74	2.905	−2.66	0.010*
*BGL*	−0.65	0.265	−2.44	0.018*
*HbA1c 5yr*	0.99	0.819	1.21	0.233
*BMI*	0.04	0.179	0.21	0.831
*Biothesiometry*	0.10	0.068	1.44	0.154
*B. Delay MDs (ms)*				
*Name*	Estimate	SE	t-stat	p-value
*(Intercept)*	55.9	16.61	3.36	0.001*
*ETDRS 35*	9.45	6.125	1.54	0.128
*ETDRS 43*	−16.2	8.826	−1.83	0.072
*BGL*	2.86	0.716	4.00	0.000*
*HbA1c 5yr*	−5.04	2.248	−2.24	0.028*
*BMI*	−2.24	0.514	−4.35	<0.001*
*Biothesiometry*	0.62	0.191	3.25	0.002*

Statistically significant (p ≤ 0.05) differences are denoted with *.

### OFA total deviations and pattern deviations

**Figure 2:**
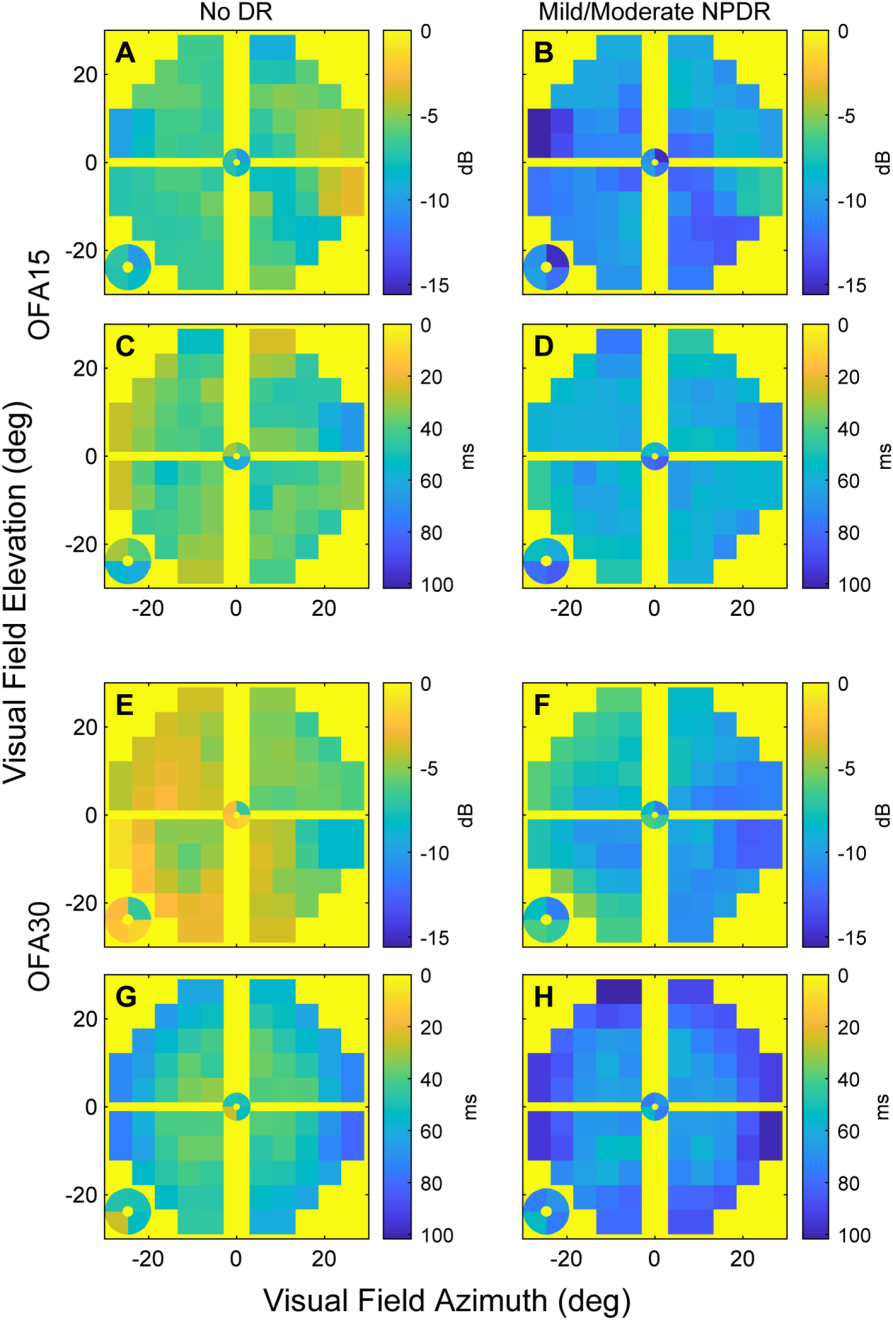
The mean sensitivity and delay TD data for OFA15 **(A-D)** and OFA30 **(E-H)**. The means were computed at each 30-2 field location across the ETDRS 10 eyes (noDR, **A, C, E, G**), and ETDRS 34 and 45 eyes (mmDR, **B, D, F, H**). From the top down, the rows alternate: sensitivities, delays, sensitivities, delays (n.b. the calibration bar units of dB and ms). Before taking the means, right-eye data were flipped to left eye format; hence, the figures have the nasal field on the right. The four small central locations are magnified and presented at the bottom left of each panel. Generally, mmDR eyes **(B, D, F, H)** showed more severe changes than those with noDR. Peripheral damage appeared to be more evident for OFA30 fields.

We next examined how the OFA data varied across the visual field. As an initial analysis, we simply took the means of the TDs across eyes in each of the two categories: noDR and mmDR (**Figure 2**). In these plots, the yellow background corresponds to the expected TD levels for normal controls, i.e., 0, cooler/darker tones represent abnormality as shown on the calibration bars. On average, the more severe eyes showed more extreme changes relative to normal. Peripheral damage was more evident for OFA30 fields (e.g., **Figure 2H**).

With that information in mind, we examined the independent effects determining the TDs using linear mixed effects models (Methods). **Table 4** shows the results for the sensitivity TDs for OFA15 (**Table 4A**) and OFA30 (**Table 4B**). We fitted the six rings of the 30-2 report (**Figure 1C**) as factors to examine the effect of visual field eccentricity, as indicated by **Figure 2**. For both models, the intercept combines the central ring 1 (**Figure 1C**), of male eyes (of the average age) and retinopathy level ETDRS 10 (noDR).

**Table 4:** Outcomes of linear mixed effects models showing the significant determinants of the OFA sensitivity total deviations (TDs) for: A) the OFA15 test, B) the OFA30 test.

A	*OFA15 sensitivities*			
*Name*	Estimate	SE	t-stat	p-value
*(Intercept)*	−6.90	0.696	−9.92	<0.001*
*Female*	−2.57	0.269	−9.54	<0.001*
*Age*	2.10	0.116	18.1	<0.001*
*Ring 2*	0.45	0.970	0.47	0.640
*Ring 3*	1.27	0.792	1.60	0.109
*Ring 4*	1.80	0.767	2.35	0.019*
*Ring 5*	1.60	0.751	2.14	0.033*
*Ring 6*	1.34	0.741	1.80	0.071
*ETDRS 35*	−3.23	0.293	−11.0	<0.001*
*ETDRS 43*	-6.42	0.400	−16.0	<0.001*
B	OFA30 sensitivities			
*Name*	Estimate	SE	t-stat	p-value
*(Intercept)*	−3.21	0.792	−4.06	<0.001*
*Female*	−0.22	0.269	−0.80	0.422
*Age*	1.77	0.118	15.0	<0.001*
*Ring 2*	−1.33	1.103	−1.21	0.227
*Ring 3*	−1.27	0.901	−1.42	0.157
*Ring 4*	−1.38	0.872	−1.58	0.113
*Ring 5*	−1.07	0.854	−1.26	0.209
*Ring 6*	−0.66	0.842	−0.79	0.432
*ETDRS 35*	−4.07	0.295	−13.8	<0.001*
*ETDRS 43*	−7.13	0.404	−17.7	<0.001*

Statistically significant (p ≤ 0.05) differences are denoted with *. The units are dB except for Age in dB/decade. The Intercept is for males of the mean age and ETDRS 10 and central Ring 1 of the 30-2 pattern (**Figure 1C**). Negative sensitivities are lower than for ETDRS 10.

For OFA15, this was −6.90 ± 0.97 dB (relative to the OFA normative data), indicating some global suppression. Response sensitivity was more suppressed in females (−2.57 ± 0.27 dB); however, it increased with age by 2.10 ± 0.12 dB/decade. ETDRS 35 and 43 further suppressed global sensitivity by around −3 dB to −6 dB. The outer rings 4 and 5 showed significant relative hypersensitivity of 1.6 to 1.8 dB (both p ≤ 0.033). Ring 6 was marginally significantly hypersensitive (p = 0.071). For OFA30, there were no significant effects of eccentricity but the outcomes for age, and ETDRS levels 35 and 45 were like those of OFA15.

**Table 5** shows the same model fitted to the OFA delay TDs. The intercepts for OFA15 and OFA30 indicated that ETDRS 10 had mean delays of 42.9 ± 3.97 ms and 35.5 ± 3.17 ms, respectively (relative to the OFA normative data). Female responses were slower than males in both tests at 11.1 ± 1.24 and 24.1 ± 1.1 ms slower, respectively (p < 0.001). Age was only significant for OFA30, increasing by 2.56 ± 0.44 ms/decade of age (p < 0.001). ETDRS 35 eyes were 22 to 27 ms slower relative to ETDRS 10 (both p < 0.001). ETDRS 43 eyes produced faster than average delays (−5.98 ± 1.73 ms) for OFA15 and slower than average for OFA30 (4.06 ± 1.51 ms), both p < 0.007. The peripheral rings 5 and 6 of OFA30 were slower by 15 ms to 23 ms relative to central ring 1 (both p < 0.001).

**Table 5:** Outcomes of linear mixed effects models showing the significant determinants of the OFA Delay Total Deviations (TDs) for: A) the OFA15 test, B) the OFA30 test.

*A*	*OFA15 delays*			
*Name*	Estimate	SE	t-Stat	p-Value
*(Intercept)*	42.9	3.97	10.8	<0.001*
*Female*	11.1	1.24	8.95	<0.001*
*Age*	−0.27	0.50	−0.55	0.584
*Ring 2*	−6.29	5.49	−1.15	0.252
*Ring 3*	−6.10	4.48	−1.36	0.173
*Ring 4*	−6.11	4.34	−1.41	0.159
*Ring 5*	−7.12	4.25	−1.67	0.094
*Ring 6*	−7.16	4.19	−1.71	0.088
*ETDRS 35*	27.6	1.27	21.7	<0.001*
*ETDRS 43*	−5.98	1.73	−3.45	0.001*
B	OFA30 delays			
*Name*	Estimate	SE	t-stat	p-value
*(Intercept)*	35.3	3.17	11.1	<0.001*
*Female*	24.1	1.12	21.7	<0.001*
*Age*	2.56	0.44	5.86	<0.001*
*Ring 2*	-2.17	4.39	-0.49	0.621
*Ring 3*	-2.82	3.59	-0.79	0.431
*Ring 4*	2.65	3.47	0.76	0.446
*Ring 5*	15.3	3.40	4.48	<0.001*
*Ring 6*	22.7	3.35	6.78	<0.001*
*ETDRS 35*	22.3	1.10	20.2	<0.001*
*ETDRS 43*	4.06	1.51	2.69	0.007*

Statistically significant (p ≤ 0.05) differences are denoted with *. The units are ms except for Age in ms/decade. The Intercept is as for **Table 2**. Positive delays are longer than for ETDRS 10.

We fitted the same models to the PDs. These showed few interesting significant effects except for OFA30 delays whose PDs showed very similar results to the TD results for rings 5 and 6 of **Table 5** (to within 0.5 ms for each).

### Diagnostic power

**Figure 3:**
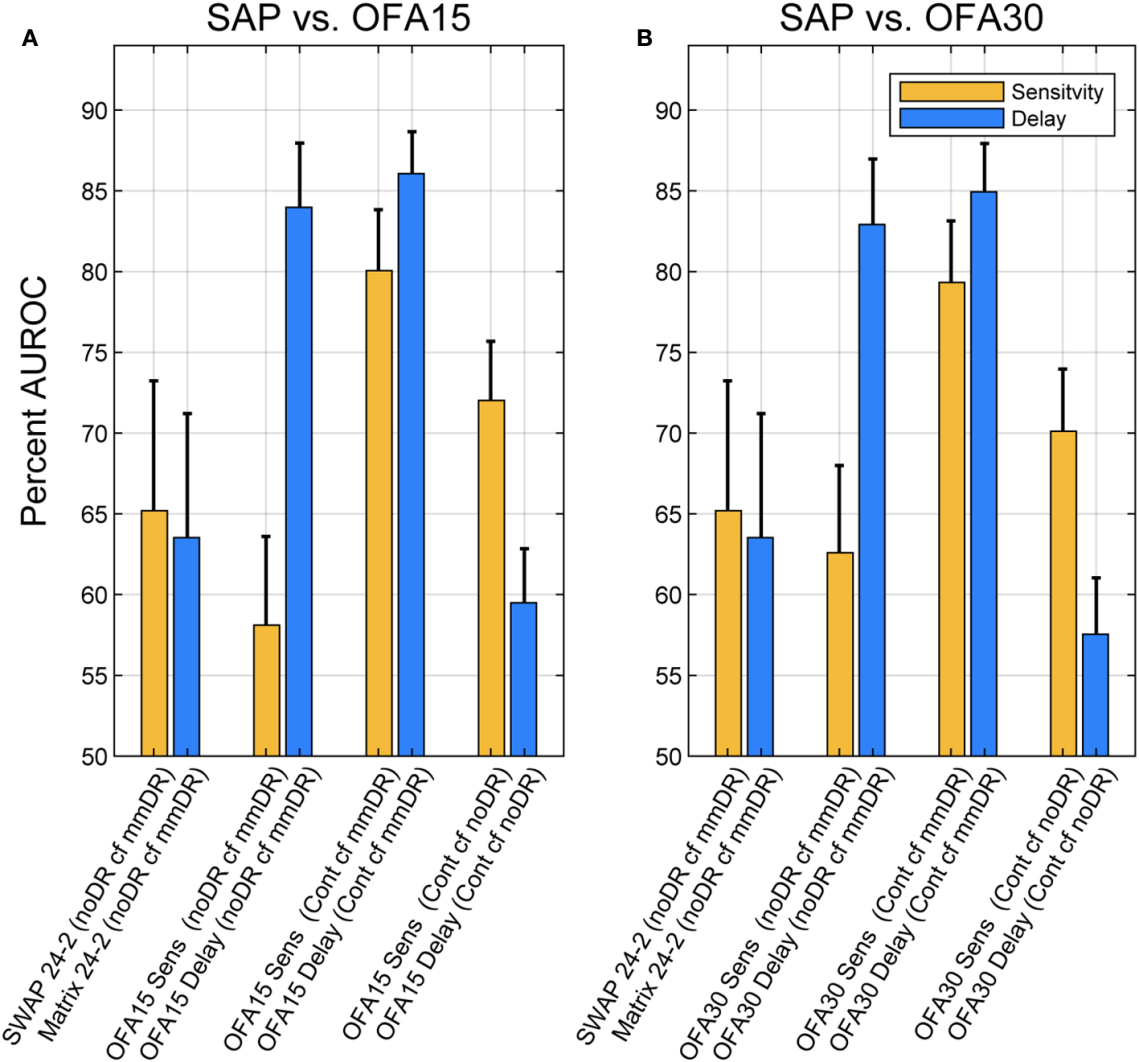
Areas under receiver operating characteristic (AUROCs) expressed as percentages for different comparisons of Total Deviations outputs. Blue bars are for delay data, and yellow are for sensitivity data (legend). AUROC of 50% represents chance classification, and an AUC of 100% represents perfect discrimination. The x-axis labels of each plot give the test: SWAP 24-2, Matrix 24-2, OFA15, or OFA30 for Sensitivities (Sens) or Times-to-peak (Delay). The leftmost four comparisons were for discriminating ETDRS 10 eyes from the Mild to Moderate NPDR eyes (ETDRS 34 and 45): (noDR cf mmDR). The rightmost four comparisons were between normal control eyes (Cont) and ETDRS 34 and 45 eyes (mmDR), or Control cf. ETDRS 10 (noDR). The leftmost pair of yellow and blue bars give AUROCs for SWAP and Matrix 24-2 tests. The SAP versus OFA15 **(A)** and OFA30 **(B)** methods performed similarly, with delays performing better except for the (Cont cf noDR) comparison where sensitivity was better. The error bars represent SEM.

To investigate the diagnostic performance of the total deviations (TDs) from the various stimulus protocols across diabetic groups, we utilized ROC analysis (Methods). **Figure 3** shows that response delay TDs (blue) were more diagnostic than those for sensitivities (yellow) especially when the discrimination was between noDR and mmDR: labeled as (noDR cf mmDR) on the x-axis. SWAP, Matrix, and OFA sensitivity-based TDs performed similarly poorly with AUROCs around 60%. OFA15 and OFA30 delays performed similarly well for the noDR/mmDR comparison at 84.0 ± 3.98% and 82.9 ± 4.06%, respectively. That was remarkable given such early disease stages were being compared. When mild/moderate eyes were compared with 85 normal control eyes from the OFA normative data (Cont cf mmDR in **Figure 3** x-axis labels), performance improved somewhat for delays at 86.1 ± 2.59% and 84.9 ± 3.00% and for OFA15 and OFA30, but more so for sensitivities at 80.0 ± 3.77% and 79.3 ± 3.81%. Interestingly sensitivities performed best for the Cont cf. mmDR comparisons relative to noDR eye comparisons.

## Discussion

To our knowledge, this study is the first report of a head-to-head comparison of both subjective and objective measures of PwT2D against structural changes in the retina and optic nerve. Our results showed significant differences between the NPDR sub-groups for OFA MDs and PSDs but not for SWAP or Matrix perimetry MDs or PSDs. Except for duration of disease, there were no differences in the clinical and demographic data of our two subgroups (**Table 1**). An earlier study using older mfPOP methods found correlations with complications screening variables like those of **Table 1** (33).

The results for OFA and Matrix are consistent with progression analysis of persons with mild DMO, where OFA metrics, but not Matrix, tracked changes in macular thickness (32, 34). A study by Montesanto et al. (15) showed significant loss relative to normal controls using Matrix; however, in our calculation using the published data, the AUROC was only around 60% (31). Other reports have shown no significant change in SWAP mean deviations in PwT2D (8, 42). A study with an achromatic Medmont perimeter showed that persons with no to mild NPDR but who had peripheral neuropathy showed statistically significant peripheral visual field loss (43).

As in previous OFA studies of diabetes (9, 11, 32, 44), peripheral retinal features like peripheral hypersensitivity and delay changes were evident (**Tables 4**, **5**). Strictly, the hypersensitivity for rings 4 to 6 for OFA15 (**Table 4**) were relative to the intercept, but in those rings of OFA15 an average of 1.28 regions/field were flagged as hypertensive at p ≤ 0.05, but only for ETDRS 10 and 35 eyes. The TDs in those regions averaged +15.0 ± 1.9 dB. These features are seen in both early-stage diabetic eye damage and age-related macular degeneration (AMD) when measured by multifocal VEPs and mfPOP on the same day in diabetes and AMD (44). Modestly hypersensitive regions have also been observed in three OFA studies of early-stage AMD (40, 45, 46). In exudative AMD, those OFA peripheral measures can also predict good outcomes from (47), or the need for (43), anti-vascular endothelial growth factor (anti-VEGF) treatment. Thus, peripheral hypersensitivity may be a feature of the early development of retinal diseases more generally. PwT2D have been reported to have MD values around 3 to 4 dB on SWAP perimetry (8). Unfortunately, unlike OFA, no form of standard automated perimetry reports the significance of any regions of hypersensitivity. In addition, the patchy distribution of damage is consistent with the observed per-region changes being afferent defects as we have shown before (9). Previous OFA studies of diabetes had reported features suggestive of peripheral hypersensitivity (9, 11, 32, 44) due to observation of faster than normal delays peripherally (32). Both may be markers for earlier-stage disease.

An interesting feature of the per-region delays was that the more peripheral rings of the larger OFA30 stimuli showed delays that were 15 to 22 ms slower (p < 0.001) than the inner rings. The inner rings of OFA30 correspond to the whole of the OFA15 stimulus, and OFA15 showed no significant delays as a function of stimulus ring. Iris neuropathy would mimic a global change in delay, i.e., the same change for all test regions. The slower responses to stimuli applied to rings 5 and 6 of OFA30 cannot therefore be due to iris neuropathy given that OFA15 and OFA30 were tested on the same day and in randomized order. It is possible that some of the effects of ETDRS 35 and 43 could be attributed to iris neuropathy but in the case of OFA15, ETDRS 43 eyes were 5.98 ± 1.73 ms quicker than the normative data (p = 0.001), whereas in the same eyes on the same day, they were slower for OFA30 by 4.06 ± 1.51 ms (p = 0.007). Here, we report (**Table 5**) that for the two stimuli, female PwT2D produced longer response delays than males by 11. 1 ± 1.24 ms (p < 0.001) and 24.1 ± 1.12 ms (p < 0.001). This may be attributed to the small sample size. Among the 85 control subjects, we took the per-subject means (giving one delay per subject) and a simple linear model indicated that males had 19.9 ± 5.09 ms (mean ± SE) longer response delays (p = 0.0002). Thus, this may be a real effect that requires further study.

For OFA30, biothesiometry was mildly associated with an increased delay MD (p = 0.002, **Table 3B**) but was not associated with changed sensitivity (**Table 3A**). Biothesiometry readings were not associated with either sensitivity or delay MD changes for OFA15 (not shown). BMI similarly reduced delay by −2.24 ms per BMI unit (p < 0.001). eGFR was not significant for sensitivity or delay for either test (not shown). Overall, metabolic/tissue-damage variables that might be linked to iris neuropathy tended not to add to global delays. By contrast, large changes in global sensitivity and delay were associated with ETDRS 10 (the intercept in **Tables 3**–**5**).

As in previous OFA studies of early-stage diabetic eye damage, AUROCs were high (9, 11) and focal changes in response delays were among the most informative measures. Those are correlated with changes in retinal thickness in DMO (32). Here, none of the SWAP, Matrix, or OFA sensitivities produced useable diagnostic power when the noDR and mmDR groups were compared (**Figure 3**). When compared with normal controls, sensitivities were almost as effective as delays. A recent review of 44 functional and structural measures from 23 studies, which examined diagnostic power for discriminating noDR eyes from control eyes using a range of structural and functional methods, found median AUROCs around 89% for OFA and 60% to 70% for the other methods (p < 0.0001). That review included a recent OFA study of young persons with type 1 diabetes, and the overall median value across nine measures from four OFA studies was 89% (31). That included a recent study using new fifth-generation OFA stimuli which test both eyes in <90 s (48). Similar results using the same method have been published for early-stage AMD (49). That rapid test is ideal for testing children and infirm persons.

The high diagnostic power of OFA methods may mean they could be useful for managing DR with newer treatments. Candesartan shows promise in the prevention of earlier-stage retinopathy in T2D patients (20). Fenofibrate is also gaining recognition as a therapy with potential to prevent progression and even reverse earlier stages of DR in T2D (18, 19). Clearly, additional new treatments that target earlier stages of diabetic eye disease are needed and these will need monitoring tools for preclinical early-stage disease and new clinical endpoints. Functional measures are more likely than structural to be able to provide surrogate endpoints that are acceptable to regulatory authorities.

The limitations of our study were the relatively small number of subjects with mild/mod NPDR. Also, patients may have been fatigued during SAP testing due to the series of tests that were conducted at each visit, which may have affected reliability of those tests. Furthermore, OFA test reproducibility was not examined in this study; however, good reproducibility of mfPOP has been demonstrated previously in T2D (9) and glaucoma (50).

The results of this study demonstrate the utility and advantage of mfPOP in detecting changes in visual function in T2D. This study is the first to complete a direct comparison between objective vs. subjective measures of visual field sensitivity corresponding to varying retinopathy severities. Small-scale longitudinal studies suggest the findings of our cross-sectional studies translate into ability to track disease progression (32, 34), with similar results in AMD (43). With the advent of novel interventions in early DR, more sensitive methods for identifying eyes at risk of progression to the sight threatening stages of DR are needed. The ability to test children with the same rapid tests that adults use (48) might assist in the management of T1D.

## Data availability statement

The raw data supporting the conclusions of this article will be made available by the authors, without undue reservation.

## Ethics statement

The studies involving humans were approved by ACT Health Human Research Committee (eth.7.07.667). The studies were conducted in accordance with the local legislation and institutional requirements. The participants provided their written informed consent to participate in this study.

## Author contributions

FS: Conceptualization, Data curation, Formal Analysis, Writing – original draft, Writing – review & editing. JK: Formal Analysis, Writing – review & editing. RI: Data curation, Supervision, Writing – review & editing. CC: Data curation, Methodology, Writing – review & editing. CN: Conceptualization, Data curation, Supervision, Writing – review & editing. RC: Data curation, Writing – original draft. TM: Conceptualization, Formal Analysis, Writing – review & editing.
